# Impact of Charge-Transfer Excitons on Unidirectional Exciton Transport in Lateral TMD Heterostructures

**DOI:** 10.1021/acs.nanolett.5c02447

**Published:** 2025-07-15

**Authors:** Roberto Rosati, Sai Shradha, Julian Picker, Andrey Turchanin, Bernhard Urbaszek, Ermin Malic

**Affiliations:** † Department of Physics, 9377 Philipps-Universität Marburg, Renthof 7, D-35032 Marburg, Germany; ‡ mar.quest|Marburg Center for Quantum Materials and Sustainable Technologies, Hans-Meerwein-Straße 6, D-35032 Marburg, Germany; ¶ Institute of Condensed Matter Physics, 26536 Technische Universität Darmstadt, 64289 Darmstadt, Germany; § Institute of Physical Chemistry, 9378 Friedrich Schiller University Jena, 07743 Jena, Germany; ∥ 27087 Abbe Centre of Photonics, 07745 Jena, Germany

**Keywords:** lateral heterostructures, charge-transfer excitons, unidirectional exciton drift, exciton trapping

## Abstract

Lateral heterostructures built of monolayers of transition-metal dichalcogenides host a thin one-dimensional interface exhibiting a large energy offset. Recently, the formation of spatially separated charge-transfer (CT) excitons at the interface has been demonstrated, but their impact on technologically important exciton propagation across the interface has remained in the dark. In this theoretical work, we microscopically investigate the spatiotemporal exciton dynamics in the exemplary hBN-encapsulated WSe_2_–MoSe_2_ lateral heterostructure. We reveal a highly interesting interplay of energy-offset-driven unidirectional exciton drift across the interface and efficient capture into energetically lower CT excitons at the interface. This interplay triggers a counterintuitive thermal control of exciton transport with less efficient propagation at lower temperatures, opposite to conventional semiconductors. We predict clear signatures of this intriguing exciton propagation in both far- and near-field photoluminescence experiments. Our results present an advance in the microscopic understanding of technologically relevant unidirectional exciton transport in lateral heterostructures.

## Graphical abstract

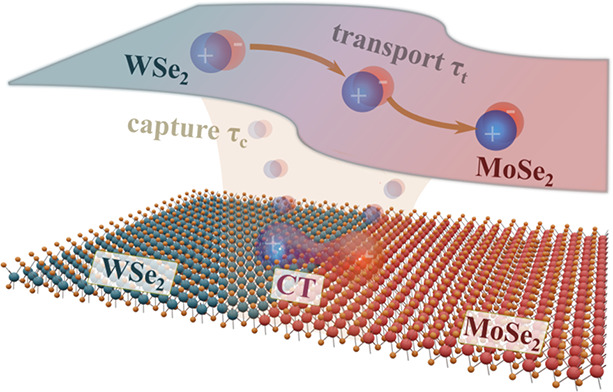

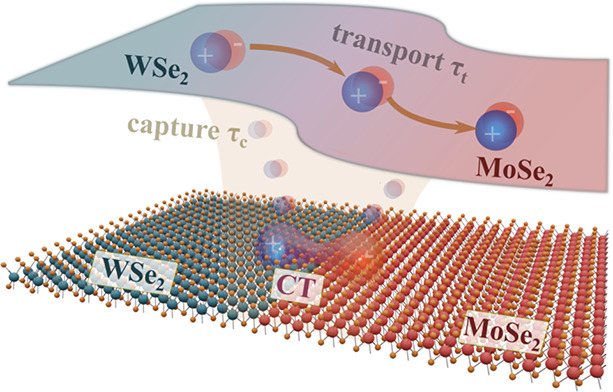

Monolayers of transition-metal dichalcogenides (TMDs) can be grown side-by-side to form lateral heterostructures (Figure 1).^1–4^ They typically exhibit a band offset at the interface, facilitating the formation of spatially separated charge-transfer (CT) excitons.^5–8^ The latter have been experimentally demonstrated^6,7^ thanks to recent developments in chemical vapor deposition (CVD) growth techniques,^3,6,9–12^ allowing narrow interface widths comparable to the exciton Bohr radius. First studies on exciton and photocarrier transport in lateral heterostructures have been performed.^7,10,13–18^ Furthermore, in near-field spectroscopy, narrow exciton distributions have been realized, allowing the observation of an unidirectional exciton drift across the interface in a WSe_2_–MoSe_2_ lateral heterostructure.^10,13^

**Figure 1:**
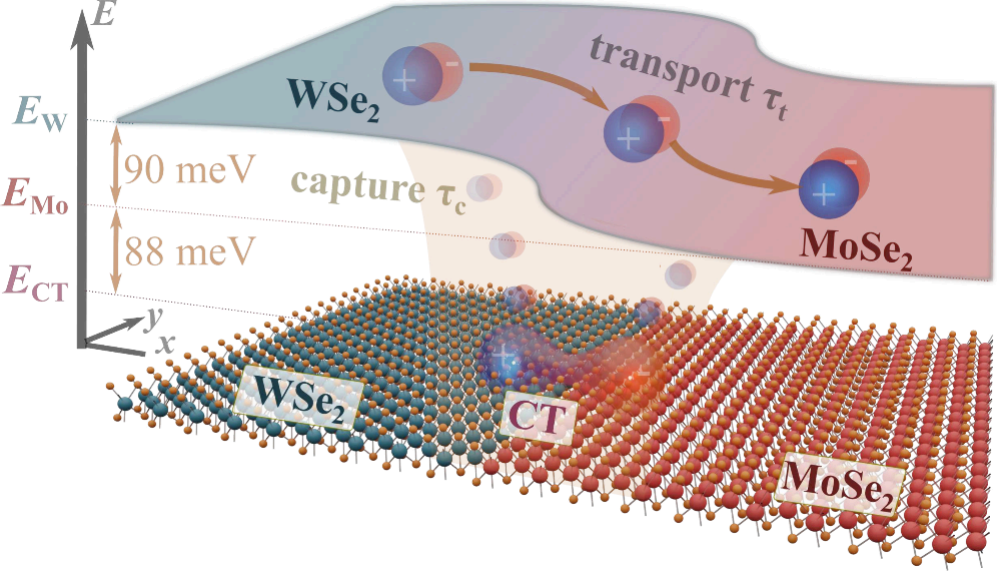
Sketch of a lateral TMD heterostructure (bottom) and the resulting spatially varying excitonic energy landscape (top). Exciton propagation is governed by the interplay of the unidirectional transport across the interface (driven by the energy offset of the WSe_2_ and MoSe_2_ excitons *E*_W_ – *E*_Mo_) and the capture into the energetically lowest CT excitons with the energy *E*_CT_.

Excitons are neutral quasiparticles, and their directional transport is challenging and has so far been mainly controlled in TMD monolayers or vertical TMD heterostructures through strain profiles^19^ or an electric field.^20^ In contrast, in lateral heterostructures, directional exciton transport occurs naturally due to the internal energy offset (Figure 1) between the bands of the constituent materials. However, the impact of CT excitons on the transport behavior has been neglected so far, although they are expected to play a crucial role when excitons propagate across the interface.

In this theoretical study, we microscopically model the spatiotemporal exciton dynamics in lateral TMD heterostructures, focusing, in particular, on the exemplary hBN-encapsulated WSe_2_–MoSe_2_ structure. We reveal an intriguing interplay between the energy-offset-driven unidirectional exciton propagation across the junction and the capture into the energetically lowest CT excitons at the interface. This results in an unexpected temperature dependence with a more pronounced exciton propagation at higher temperatures, contrary to the behavior in conventional semiconductors. We predict distinct signatures of this intriguing propagation behavior in near-field spectroscopy experiments because the crossing point in space of the photoluminescence (PL) intensity of MoSe_2_ and WSe_2_ exciton transitions is not at the lateral crystal junction but at a spatial offset Δ_X_ several hundred nanometers away from it, in excellent agreement with previously performed fully independent experimental data.^10^ Overall, our microscopic and material-specific approach sheds light on exciton propagation across the interface of technologically promising lateral TMD heterostructures, particularly emphasizing the importance of CT excitons. The gained insights can trigger and guide experimental studies of exciton transport in lateral heterostructures.

We first microscopically calculate the exciton energy landscape in the hBN-encapsulated WSe_2_–MoSe_2_ lateral heterostructure by numerically solving the Schrödinger equation. We consider an interface width of 2.4 nm, as realized in recent CVD-grown samples,^6^ and include the Coulomb interaction via a Keldysh–Rytova potential.^21,22^ We make use of the large difference between the relative and total exciton mass^23^ to separate the Schrödinger equation into two coupled equations (see the Supporting Information, SI).^5,6^ The first is a Wannier-like equation in the relative electron–hole position, determining if electrons and holes are bound. The solution enters the second equation, which provides an additional quantization in the center-of-mass position.^5,6^ This leads to the formation of CT excitons at the interface. They exhibit an energy *E*_CT_ that is clearly lower than the energy of MoSe_2_ and WSe_2_ excitons *E*_Mo_ and *E*_W_ (Figure 1). The considered heterostructure has a spatial energy offset of *E*_W_ – *E*_Mo_ = 90 meV^10^ at the interface, and CT excitons are found to be 88 meV below *E*_Mo_.^6^ This energy offset is expected to drive the transport of excitons from the WSe_2_ side toward the energetically favorable MoSe_2_ side. While excitons are crossing the interface, they are likely to be trapped in the energetically lower CT exciton states (Figure 1).

Along the interface, there is no energy offset, resulting in a driftless diffusion,^7,24^ while the offset-driven propagation across the interface can be described via one-dimensional Wigner functions *f*_α_(*x*,*q*_*x*_,*t*) = 1/*L*_*y*_∫∑_*q*_*y*__∑_**q**′_⟨*X̂*_α,**q**+**q**′/2_^†^ *X̂*_α,**q**–**q**′/2_⟩e^ı**q** ^′^·**r**^ d*y*. Here, *x* and *q*_*x*_ are the components across the interface (which is set to be along the *y* direction) of the center-of-mass position **r** = (*x*, *y*) and momentum **q** = (*q*_*x*_, *q*_*y*_), while *L*_*x*,*y*_ is the system length across/along the interface. The index α = W and Mo refers to excitons at the WSe_2_ and MoSe_2_ sides of the heterostructure, respectively, while *X̂*_α,**q**_^(†)^ are exciton annihilation (creation) operators.^25,26^ Using the Heisenberg equation of motion and introducing the reduction to one dimension, we derive the spatiotemporal dynamics of the one-dimensional Wigner function, yielding (see the SI)­

1 $${ḟ}_{\alpha }=-v_{q_{x}}\;\frac{\partial }{\partial_{x}}f_{\alpha }+\frac{f_{\alpha }^{{}^{\circ}}-f_{\alpha }}{\tau_{p}}+{ḟ}_{\alpha }^{d}+{ḟ}_{\alpha }^{\mathrm{cap}}$$

The first term describes the regular exciton propagation driven by the group velocity *v*_*q*_*x*__ = ℏ*q*_*x*_/*M* (with the total exciton mass *M*) and the gradient in the occupation. The second term describes the phonon-driven thermalization toward the local Boltzmann distribution *f* _α_^°^. This is modeled with a relaxation-time approximation with the microscopically obtained phonon-driven scattering rate τ_p_.^27^ As the initial exciton occupation, we choose a Gaussian distribution centered at the interface or varied along the heterostructure (see the SI for more details).

While the first two contributions are also present in regular TMD monolayers, the third and fourth terms in eq 1 are specific to lateral TMD heterostructures. The third term describes the drift of excitons driven by the energy offset at the interface and reads

2 $${ḟ}_{\alpha }^{d}\left(x,q_{x},t\right)=\underset{q_{x}^{\prime }}{\sum }V\left(x,q_{x}-q_{x}^{\prime }\right)\;f_{\alpha }\left(x,q_{x}^{\prime },t\right)$$

The spatial variation of exciton energies *E*_α_(*x*) induces the superpotential $V\left(x,q_{x}\right)$ defined as

$$V=\frac{-\iota }{\hbar L_{x}}\int_{-x_{V}}^{x_{V}}\;{dx}^{\prime }\;\left[E_{\alpha }\left(x+\frac{x^{\prime }}{2}\right)-E_{\alpha }\left(x-\frac{x^{\prime }}{2}\right)\right]e^{-\iota q_{x}x\prime }$$

which corresponds to the regular semiclassical drift 1/ℏ∂_*x*_ *E*_α_(*x*)∂_*q*_*x*__ *f*_α_ for smooth spatial variations of *E*_α_ (see the SI).^28^ Finally, the capture of optically excited MoSe_2_ and WSe_2_ excitons into the energetically lower-lying CT exciton states during the propagation across the interface is described by the fourth term in eq 1, reading

3 $${ḟ}_{\alpha }^{\mathrm{cap}}=\frac{\Delta n_{\alpha }\;f_{\alpha }^{{}^{\circ}}-f_{\alpha }}{\tau_{c}}$$

Here, we have introduced $\Delta n_{\alpha }=\frac{n_{\alpha }^{{}^{\circ}}\left(x,t\right)}{n_{\alpha }\left(x,t\right)}$ as the ratio between the spatiotemporal exciton density *n*_α_(*x*,*t*) = 1/*L*_*x*_∑_*q*_*x*__ *f*_α_(*x*,*q*_*x*_,*t*) and its thermalized analogous *n*_α_^°^(*x*,*t*) after the introduction of CT excitons. The capture process is driven by the emission of optical phonons^29–31^ with microscopically calculated scattering times τ_c_ (see the SI).^27^ The consequence is a decrease of WSe_2_ and MoSe_2_ exciton density *n*_α_ and the buildup of a CT exciton density *n*_CT_(*x*,*t*) at the interface. Note that the capture is strongly temperature-dependent via local thermalized exciton density *n*_α_^°^. At higher temperatures, the efficiency of the capture process is reduced because excitons can escape by the absorption of phonons.^30^

The knowledge of exciton densities allows to microscopically model spatially and temporally resolved PL, which is determined by a product of the exciton density and the oscillator strength of the involved exciton species. The PL intensity is described by an Elliot formula,^32,33^ which we have generalized to include CT excitons^6^

4 $$I\left(E,x,t\right)=\underset{\alpha }{\sum }\frac{f_{\alpha }^{{}^{\circ}}\left(x,q=0,t\right)\;{\mathrm{\gamma ̃}}_{\alpha }\left({\mathrm{\gamma ̃}}_{\alpha }+\Gamma \right)}{{\left(E-E_{\alpha }\right)}^{2}+{\left({\mathrm{\gamma ̃}}_{\alpha }+\Gamma \right)}^{2}}$$

with the exciton index α = W and Mo and CT and *f* _α_^°^ as the corresponding equilibrium exciton distribution. Furthermore, the PL is influenced by the temperature-dependent exciton–phonon dephasing rate^27^ Γ = ℏ/2τ_p_ and the radiative decay rate γ̃ = γ_α_|ψ_α,**q**=0_|^2^. Here γ_α_ is the oscillator strength, which is proportional to the probability of finding electrons and holes at the same position. This is about 35 smaller for spatially separated CT excitons compared to regular MoSe_2_ and WSe_2_ excitons,^6^ due to the dipole which, furthermore, has influence on the binding energy in analogy to the case of interlayer excitons.^6,34^ The momentum conservation implies that only the **q** = 0 component of the exciton wave function ψ_α,**q**_ allows radiative recombination.

In TMD monolayers, localized exciton densities propagate in space preserving their shape, which at low excitations only becomes spatially broader due to diffusion.^35^ Exciton drift can be obtained by strain engineering^19,36–41^ or by applying electric fields in the case of vertical TMD heterostructures, where dipolar interlayer excitons are involved.^20,42,43^ In lateral TMD heterostructures, the transport behavior is drastically different, with qualitative changes in the shape of the propagating exciton densities as a result of an intriguing interplay of energy-offset-driven unidirectional exciton transport across the junction and capture into energetically lower CT excitons at the interface.

We investigate the exciton transport in hBN-encapsulated WSe_2_–MoSe_2_ lateral heterostructures by numerically evaluating eq 1, which explicitly takes into account the interplay of drift and capture processes. Figure 2 shows the spatially and temporally resolved densities of (a and c) MoSe_2_ and WSe_2_ excitons as well as of (b and d) CT excitons after optical excitation at the interface (laser position at *x*_L_ = 0 nm) with a typical diffraction-limited spot [full width at half-maximum (fwhm) of 1 μm]. The excitation is set to be resonant to the WSe_2_ exciton and creates an initially broad spatial exciton density *n*_W_ at the WSe_2_ side of the lateral heterostructure, with a small but finite nonresonant occupation of MoSe_2_ excitons (about 100 times smaller than that for WSe_2_),^44,45^ (cf. the blue line in Figure 2c). The results are robust against different excitation conditions, with only minor quantitative changes depending on the energy and localization of the laser pulse (see the SI for more details). The initial shape changes rapidly because the energy offset *E*_W_ – *E*_Mo_ = 90 meV at the interface gives rise to a drift of excitons toward the energetically more favorable MoSe_2_ side (Figure 1). This unidirectional transport leads to an accumulation of the exciton density at the MoSe_2_ side of the interface (cf. the red line in Figure 2c). The exciton accumulation becomes visible after a few tens of picoseconds, and it extends over hundreds of nanometers into the MoSe_2_ side. Such broad spatial extensions are already observable in the experimental setups with a spatial resolution of a few hundreds of nanometers.^7,10,19,35,46,47^

**Figure 2:**
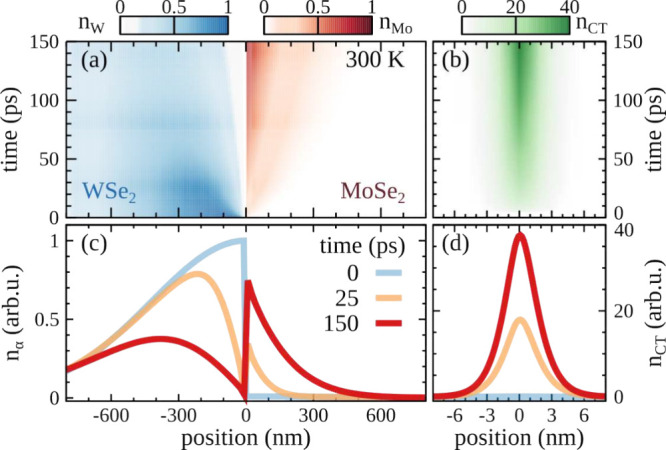
Space- and time-resolved densities of (a) WSe_2_ and MoSe_2_ excitons as well as of (b) CT excitons at 300 K after optical excitation at the interface (*x*_L_ = 0) resonant with *E*_W_. (c and d) 2D cuts of exciton densities at given times. The large CT exciton occupation and the exciton accumulation at the MoSe_2_ side of the interface can be traced back to the interplay between the unidirectional exciton drift across the interface and capture into CT excitons.

During unidirectional propagation across the interface, excitons can become trapped in the energetically lower-lying CT excitons. This results in a local depletion of the exciton density *n*_W_ at the interface (cf. the orange and red lines in Figure 2c). At the same time, the density of CT excitons considerably increases (Figure 2b,d). Its maximum becomes almost 2 orders of magnitude larger than the excited density within the first 150 ps. The efficient trapping into CT excitons has important fundamental and technological applications. Because CT excitons are dipolar and repel each other, the quick increase of their density can explain the recently observed nonlinear, dipole-driven CT-exciton diffusion along the interface.^7^ Because CT excitons have a small binding energy of a few tens of millielectronvolts,^6^ the trapping of WSe_2_ or MoSe_2_ excitons could lead to their efficient dissociation, which is favorable for many optoelectronic devices.

In a nutshell, the drift-induced unidirectional propagation results in an exciton accumulation at the low-energy side of the interface, and the efficient capture processes give rise to large CT exciton densities. The formation time and the height of exciton accumulations are determined by the interplay of exciton capture and drift, and their competition can be thermally controlled.

Carrier drift becomes generally faster at lower temperatures because here the efficiency of scattering with phonons is decreased. Interestingly, we find the opposite behavior for exciton propagation in the lateral TMD heterostructures. Figure 3a illustrates the temperature-dependent spatiotemporal dynamics of the MoSe_2_ and WSe_2_ excitons. The height of the drift-induced exciton accumulation at the MoSe_2_ side becomes surprisingly smaller with decreasing temperature *T* (and vanishes at cryogenic temperatures below 100 K, see the SI); i.e., the unidirectional transport becomes weaker at lower *T*. This behavior can be traced back to the efficient trapping process of CT excitons at the interface. It suppresses the drift by capturing the propagating WSe_2_ excitons before they reach the MoSe_2_ side. To show this, we consider the case without CT excitons (corresponding to lateral heterostructures with larger interface widths^6^). Here, we find the expected temperature trend, i.e., a faster propagation toward the low-energy side at smaller temperatures (Figure 3b). Note that the accumulation is overall 1 order of magnitude higher without CT excitons. The larger impact of the capture at smaller temperatures is due to a suppression of the counteracting escape processes driven by phonon absorption. Besides temperature, interface and dielectric engineering also can be used as externally accessible tuning knobs to control the unidirectional exciton transport across the interface. Smaller energy band offsets and/or dielectric constants of the surrounding substrate reduce the energy separation between monolayer and CT excitons,^6^ giving rise to a less efficient exciton capture at the interface and thus faster unidirectional exciton transport, as discussed in detail in the SI.

**Figure 3:**
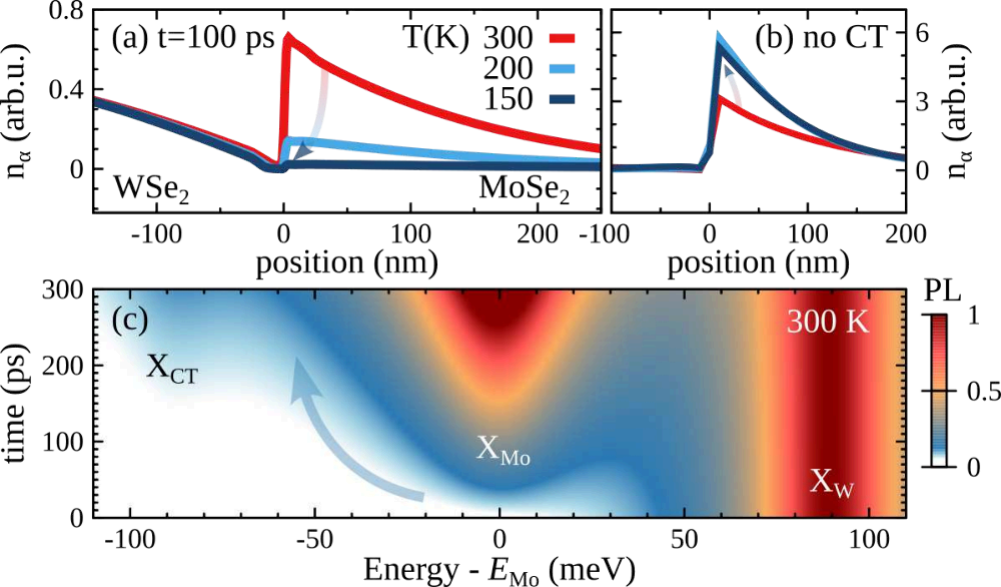
(a) Temperature-dependent accumulation of exciton density after 100 ps. The decrease as a function of temperature is opposite to the behavior in conventional semiconductors, which we obtain when (b) CT excitons are switched off. (c) Space-integrated and time-resolved PL spectrum (normalized to the intensity of X_W_), with a delayed formation of the X_Mo_ resonance. The latter is due to the energy-offset-driven propagation across the interface.

The exciton accumulation at the MoSe_2_ side after a resonant excitation of WSe_2_ excitons is a hallmark of unidirectional exciton transport. This can be revealed in space-integrated and time-resolved PL (Figure 3c). The spectrum initially shows only one peak X_W_ stemming from optically excited WSe_2_ excitons centered at *E*_W_. The energy-offset-induced exciton drift results in the formation of an additional resonance at *E*_Mo_ after a certain time delay. While the line width of this peak is fixed by radiative-recombination and phonon-mediated scattering,^27^ its height relative to X_W_ increases in time due to the unidirectional exciton propagation. At room temperature, X_Mo_ becomes as intense as X_W_ after approximately 250 ps. The situation is drastically different at smaller temperatures, where the capture-induced suppression of exciton propagation results in a negligible intensity of the drift-driven X_Mo_ resonance (see the SI). Note that an excitation resonant to *E*_Mo_ does not result in the formation of a X_W_ peak because excitons would need to increase their energy by about 90 meV. This indicates the possibility of an optical control of the unidirectional exciton drift that is potentially interesting for excitonic diodes.^10,48^ The exciton drift can be quantified in space-integrated experiments by the relative peak difference Δ*I* = (*I*_W_ – *I*_Mo_)/(*I*_W_ + *I*_Mo_). Furthermore, we derive an effective drift velocity *v*_eff_ ≈ $\Delta_{0}\sqrt{\pi /8}\left(1+I_{\mathrm{Mo}}/I_{W}\right)\partial_{t}\Delta I$ , with Δ_0_ being the width of the initial Gaussian. Surprisingly, we find that, at room temperature, *v*_eff_ is almost independent of the excitation conditions (laser energy or spot size), averaging to a value of about 1 nm/ps (see the SI for more details).

Surprisingly, we also observe the appearance of a small CT exciton peak X_CT_ after a few hundreds of picoseconds even at room temperature (Figure 3c). The peak becomes more intense at reduced temperatures, in particular, compared to the X_Mo_ resonance. The formation time of the CT exciton resonance is, however, close to typical PL decay times of 150–200 ps in WSe_2_–MoSe_2_ lateral heterostructures.^9,10^ The predicted CT exciton signal corresponds only to a few percent of the total time-integrated PL. As a consequence, such a small CT exciton peak that is clearly present in the time-resolved PL spectra might be difficult to resolve in time-integrated, energy-resolved PL, in agreement with previous studies.^6,10^ Thus, we investigate now optical excitation in the nanometer range, as can be realized in near-field spectroscopy.

In near-field scanning microscopy, we focus on excitation spots with a fwhm of 50 nm and varying positions in space. In this way, we create asymmetric MoSe_2_ and WSe_2_ exciton total occupations, as realized in recent experiments.^10,13,49^ Figure 4a shows the relative space- and time-integrated PL intensities as a function of the laser position across the interface. We consider an exciton decay time of 175 ps, as extracted from measurements on the same lateral heterostructure.^10^ Upon excitation at the MoSe_2_ side, the PL is, as expected, clearly dominated by *I*_Mo_ and the *I*_W_ signal is negligible (cf. the blue and red lines in Figure 4a).

**Figure 4:**
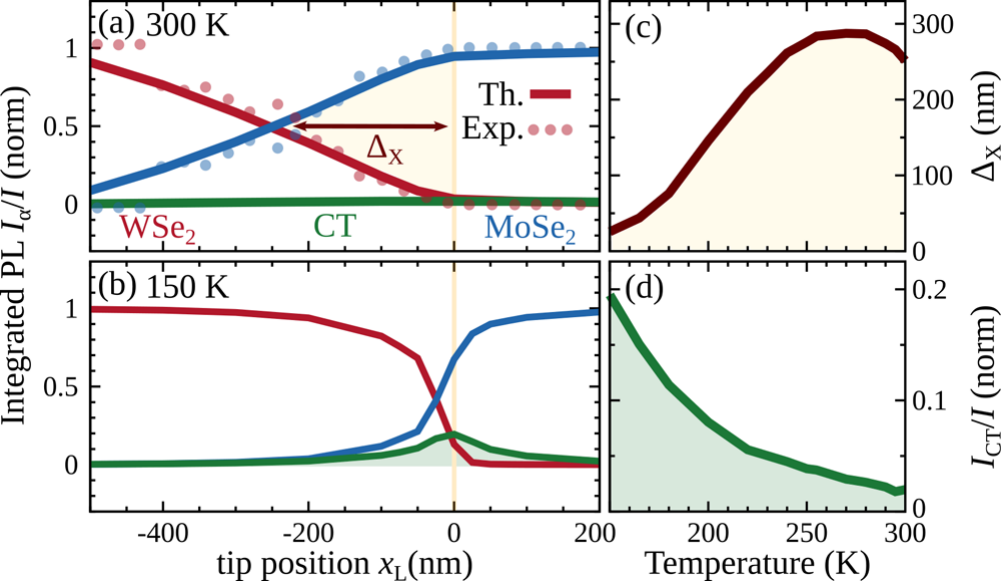
Intensity of the X_Mo_, X_W_, and X_CT_ resonances in PL spectra at (a) 300 and (b) 150 K after a near-field nonresonant excitation at different laser positions (normalized to the total intensity *I*). The vertical yellow line indicates the position of the interface. The dots show the corresponding experimental measurement taken from ref 10. The interplay of the energy-offset-driven unidirectional exciton propagation and capture into CT excitons breaks the symmetry resulting in a spatial offset Δ_X_ from the interface, at which the emission from WSe_2_ and MoSe_2_ excitons is equal, in excellent agreement between theory and experiment. (c and d) Temperature-dependent offset Δ_X_ and CT exciton emission *I*_CT_/*I*. Experimental data in part a adapted and renormalized from ref 10. Available under a CC-BY 4.0. Copyright 2022 The Authors.

Naively, we would expect excitations on the WSe_2_ side to induce PL dominated by the X_W_ resonance. The predicted PL is, however, drastically different due to the energy-offset-driven unidirectional transport toward the MoSe_2_ side and the capture processes into CT excitons at the interface. When the excitation spot is far away from the interface (>500 nm), the PL behaves as expected and *I*_W_ dominates. However, for excitation closer to the interface (<250 nm), *I*_Mo_ becomes more pronounced than *I*_W_. Note that this occurs for excitation spots with a distance 5 times larger than the excitation confinement of 50 nm. The reason for this behavior is the efficient exciton drift, which drives the excited WSe_2_ excitons toward the energetically more favorable MoSe_2_ side. The drift efficiency can be quantified by the spatial offset Δ_X_ from the interface providing an equal PL intensity from MoSe_2_ and WSe_2_ excitons. Without a drift, we found Δ_X_ to be 0, as expected. At room temperature, we predict a value of Δ_X_ ≈ 250 nm. This is in excellent agreement with recent near-field experiments,^10,13^ (cf. the dots in Figure 4a).

Given the thermal control of exciton transport discussed above, we investigate the near-field PL at a lower temperature of 150 K (Figure 4b). We observe that the spatial offset becomes smaller with Δ_X_ ≈ 25 nm, reflecting the capture-induced suppression of the exciton drift at decreasing temperatures (see also Figure 3a). This is confirmed by a temperature study in Figure 4c, where we interestingly also find a slight decrease of Δ_X_ for temperatures from 280 to 300 K. This stems from the competition between exciton capture and exciton drift, which show an opposite temperature dependence (see the SI). The efficient capture is further demonstrated in the increasing PL intensity of CT excitons at lower temperatures (Figure 4d). Here, the near-field excitation allows one to visualize the CT resonance even in time-integrated PL.

On the basis of a microscopic, material-specific, and predictive approach, we have studied exciton transport in lateral TMD heterostructures. We demonstrate a pronounced unidirectional exciton drift due to the energy offset across the interface, resulting in an accumulation of excitons at one side of the interface. Furthermore, we predict a crucial impact of capture processes on the energetically lower-lying CT excitons at the interface. Finally, we demonstrate that temperature is the key knob to control the interplay between unidirectional exciton drift and the capture-induced formation of CT excitons. Our theoretical results are confirmed by previous independent experiments^10^ and can guide future measurements on spatiotemporal exciton dynamics. On the basis of the gained microscopic knowledge, we provide concrete recipes for detecting the intriguing exciton propagation in both far- and near-field PL experiments. Our findings also have potential technological importance for devices based on unidirectional exciton transport in lateral heterostructures.

## Supplementary Material



## Notes

Overview of the theoretical approach and the derivation of the one-dimensional reduction of the two-dimensional spatiotemporal dynamics, case of nonresonant excitation, and dielectric, interface, and excitation engineering (PDF)
