# Supplementary material for: Impact of Charge-Transfer Excitons on Unidirectional Exciton Transport in Lateral TMD Heterostructures
Source: Nano Lett. 2025 Jul 15;25(29):11319–24. doi: 10.1021/acs.nanolett.5c02447 (PMC12291580; doi:10.1021/acs.nanolett.5c02447)
Supplement: Supplementary file 1 [file nl5c02447_si_001.pdf]

Supplementary Information for Impact of Charge Transfer Excitons on Unidirectional Exciton Transport in Lateral TMD Heterostructures

Roberto Rosati,[1,2,*] Sai Shradha,[3] Julian Picker,[4,5] Andrey Turchanin,[4,5] Bernhard Urbaszek,[3] and Ermin Malic[1,2]

[1]*Department of Physics, Philipps-Universität Marburg, Renthof 7, D-35032 Marburg, Germany*
[2]*mar.quest | Marburg Center for Quantum Materials and Sustainable Technologies, Hans-Meerwein-Straße 6, D-35032 Marburg, Germany*
[3]*Institute of Condensed Matter Physics, Technische Universität Darmstadt, 64289 Darmstadt, Germany*
[4]*Institute of Physical Chemistry, Friedrich Schiller University Jena, 07743 Jena, Germany*
[5]*Abbe Centre of Photonics, 07745 Jena, Germany*

1. EXCITON ENERGY LANDSCAPE

We investigate lateral heterostructures (LHs) based on transition metal dichalcogenide monolayers (TMD). We solve the Schrödinger equation including the space-dependent energy landscape due to the presence of an interface, cf. Fig. S1. The two different materials induce an in-plane variation of energy, which we describe via the single-particle band-edge energies $E^0_{c/v}(\mathbf{r}_{c/v})$ of conduction- and valence-band electrons, respectively, with $\mathbf{r}_{c/v}$ being their respective positions. These single-particle energies read $E^0_{c/v}(\mathbf{r}_{c/v}) = \Delta E_{c/v}(1 - \tanh(4x_{c/v}/w))/2 + E^0_{\rm Mo}(1 \pm 1)/2$ with $+$ $(-)$ for $c\,(v)$, cf. Fig. S1(b) [1, 2]. Here, w denotes a finite width of the interface, reflecting the typical situation in real samples due to spontaneous alloying [3, 4]. In particular, we consider a narrow interface of w=2.4 nm, as recently realized experimentally [2]. While lateral heterostructures involving TMDs with different chalcogen atoms have a lattice mismatch, in WSe_2-$MoSe_2$ we can assume a strain-free interface [5, 6]. The conduction and valence bands form an offset $\Delta E_c, \Delta E_v$ at the interface, typically inducing a type-II alignment [7–11]. In the specific case of WSe_2-$MoSe_2$ LH, the conduction band minimum is located in the $MoSe_2$ layer [12], hence only the bright valley is relevant for charge transfer (CT) excitons in WSe_2-$MoSe_2$ [2]. We take a valence-band offset ΔE_v=215 meV in agreement with the microscopic estimation in similar LHs [12]. In addition, the two materials exhibit also a different bandgap with $E^0_{\rm W} - E^0_{\rm Mo} = 90$ meV [13] leading to $\Delta E_c = \Delta E_v + 90$ meV. The different bandgap at the two sides of the heterostruture is different than in the case of gate-induced homojunctions [14–16], where the band offsets are the same ($\Delta E_c = \Delta E_v$) and where bound excitons confined to few tens of nanometers can appear [17, 18]. The type-II alignment allows for a continuum of unbound CT electron-hole pairs, with electrons and holes stemming from different sides of the interface and forming an unbound continuum with the minimum energy $E^0_{\rm CT} = E^0_{\rm Mo} - \Delta E_v = E^0_{\rm W} - \Delta E_c$ (cf. the purple oval in Fig. S1(b)).

To investigate if also bound CT excitons can form, we include the electron-hole Coulomb interaction $V_C(\mathbf{r}_r)$, which is described by a generalized Keldysh potential [19–21] for charges in a thin-film surrounded by a spatially-homogeneous dielectric environment [19–21]. Here we have introduced the center-of-mass and relative positions $\mathbf{r} \equiv (x, y)$ and $\mathbf{r}_r$, respectively. In view of the difference between the relative and total exciton mass μ and M, respectively, the resulting Schrödinger equation can be separated in equations for the relative and the center-of-mass motion [1, 2] yielding

$$\left[\frac{-\hbar^2\nabla^2_{\mathbf{r}_r}}{2\mu} + V_C(\mathbf{r}_r) + V^x(\mathbf{r}_r)\right]\phi^x_j(\mathbf{r}_r) = \tilde{E}_j(x)\phi^x_j(\mathbf{r}_r)\,, \tag{1a}$$

$$\left[-\frac{\hbar^2}{2M}\partial^2_x + \tilde{E}_j(x)\right]\psi_{n,j}(x) = E_{n,j}\psi_{n,j}(x)\,, \tag{1b}$$

where $V^x(\boldsymbol{r}_r) = E^0_c(\boldsymbol{r}_r, x) - E^0_v(\boldsymbol{r}_r, x)$ acts as an interface potential given by the space-dependent band edges $E^0_{c,v}$ and the masses μ and M, which we include as material-specific input parameters from first-principles calculations for WSe_2 [22]. Here, Eq. (1a) is a Wannier-like equation stating if electrons and holes can bind together via a quantization in relative motion, with j =1s, 2s, 3s, ... labeling the bound states. In this work, we focus on the energetically lowest 1s excitons justified by the large energy separation of excited states. The eigenenergies $\tilde{E}_j(x)$ found by solving Eq. (1a) act as a potential in Eq. (1b), where they can induce an additional quantization in the center-of-mass position,

*Electronic address: roberto.rosati@physik.uni-marburg.de

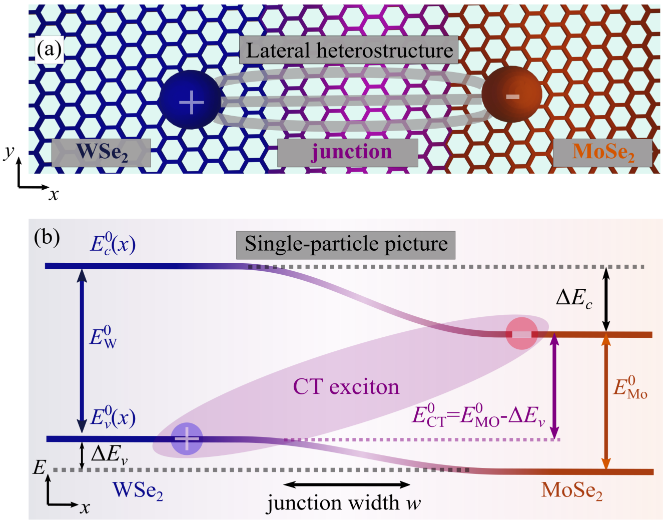

Fig. S1: (a) Sketch of a lateral WSe_2-$MoSe_2$ heterostructure. (b) The two materials have intrinsic bandgaps E^0_{Mo} and E^0_{W} and forming conduction and valence band offsets $\Delta E_c, \Delta E_v$ around the junction. Spatially-separated electrons and holes across the interface form charge-transfer (CT) excitons with the corresponding continuum energy $\mathrm{E}^0_{\mathrm{CT}} = E^0_{\mathrm{Mo}} - \Delta E_v$.

resulting in the emergence of bound CT excitons localized at the interface. In the monolayer limit of no band offsets, i.e. $\Delta E_{c/v} = 0$, Eq. (1a) becomes the well-known Wannier equation with a space-independent potential and $\tilde{E}_j(x) \equiv \tilde{E}_j$. In this limit, the center-of-mass equation (Eq. (1b)) becomes trivial corresponding to fully delocalized plane waves $\psi_n(x) \equiv \psi_{q_x}(x) = e^{\imath q_x x}$ and resulting in $E_{n,j} \equiv E_{q_x,j} = \tilde{E}_j + \hbar^2 q_x^2/2M$. Here, q_x is the component across the interface of the center-of-mass momentum $\mathbf{q} = (q_x, q_y)$. This implies that the center-of-mass motion of excitons is free and there is no quantization. For WSe_2-$MoSe_2$ LHs, we find CT excitons with an energy $\mathrm{E}_{\mathrm{CT}} \equiv E_{1,1s}$ that is approximately 88 meV lower than $\mathrm{E}_{\mathrm{Mo}} \equiv \tilde{E}_{1s}(x \gg 0)$ (and 178 meV lower than $\mathrm{E}_{\mathrm{W}} \equiv \tilde{E}_{1s}(x \ll 0)$). While spatially localized across the interface, CT excitons are delocalized along the junctions, where states with the momentum q_y along the interface form a one-dimensional parabolic dispersion. Overall, the wave-function of CT excitons reads $\psi_{q_y}(\mathbf{r}, \mathbf{r})_r = \psi_{\mathrm{CT}}(x)\, \phi^x_{1s}(\mathbf{r}_r) \frac{1}{\sqrt{L_y}} e^{\imath q_y y}$, where $\psi_{\mathrm{CT}}(x) \equiv \psi_{1,1s}$ is the ground eigenstate in Eq. (1b) and L_y is the length of the sample along the interface.

2. SPATIOTEMPORAL EXCITON DYNAMICS

To investigate the spatiotemporal exciton dynamics in a lateral heterostructure, we first introduce a two-dimensional description of the dynamics and then exploit the sample symmetry to reduce it to the simpler one-dimensional case. For this purpose, we start with the Wigner functions $f^{2\mathrm{D}}_\alpha(\mathbf{r}, \mathbf{q}, t) = \sum_{\mathbf{q}'} \langle \hat{X}^\dagger_{\alpha,\mathbf{q}+\mathbf{q}'/2} \hat{X}_{\alpha,\mathbf{q}-\mathbf{q}'/2} \rangle e^{\imath \mathbf{q}' \cdot \mathbf{r}}$ with $\alpha =$W, Mo denoting WSe_2 and $MoSe_2$ excitons, respectively. Here, $\hat{X}^{(\dagger)}_{\alpha,\mathbf{q}}$ are the exciton annihilation (creation) operators [23, 24]. Using the Heisenberg equation of motion [25], we derive the spatiotemporal dynamics of the Wigner function yielding

$$\dot{f}^{2\mathrm{D}}_\alpha(\mathbf{r}, \mathbf{q}, t) = -\, v_{\mathbf{q}} \cdot \nabla f^{2\mathrm{D}}_\alpha(\mathbf{r}, \mathbf{q}, t) + \frac{f^{2\mathrm{D},\circ}_\alpha(\mathbf{r}, \mathbf{q}, t) - f^{2\mathrm{D}}_\alpha(\mathbf{r}, \mathbf{q}, t)}{\tau_p} + \dot{f}^{2\mathrm{D},\,\mathrm{d}}_\alpha(\mathbf{r}, \mathbf{q}, t) + \dot{f}^{2D,\mathrm{cap}}_\alpha(\mathbf{r}, \mathbf{q}, t). \tag{2}$$

The first term describes the regular exciton propagation driven by the group velocity $v_{\mathbf{q}} = \hbar \mathbf{q}/M$ and the gradient in the occupation. The second term describes the phonon-driven thermalization toward the local Boltzmann distribution

$$f^{2\mathrm{D},\circ}_\alpha(\mathbf{r}, \mathbf{q}, t) = A \bar{f}^{2\mathrm{D},\circ}_\alpha(\mathbf{q})\, n_\alpha(\mathbf{r}, t) \quad , \tag{3}$$

with $\bar{f}^{2\mathrm{D},\circ}_\alpha(\mathbf{q}) = \frac{2\pi\hbar^2}{M k_B T} \exp\left(-\frac{\hbar^2 |\mathbf{q}|^2}{2M k_B T}\right)/A$ being the normalized Boltzmann distribution, where $A = L_x L_y$ is the area of the sample. Here, $n_\alpha(\mathbf{r}, t) = \frac{1}{A} \sum_{\mathbf{q}} f^{2\mathrm{D}}_\alpha(\mathbf{r}, \mathbf{q}, t)$ is the corresponding spatial exciton density. The phonon-driven thermalization is typically described by the Boltzmann collision term, which can be simplified in the relaxation-time approximation in the limit of $\sum_{\mathbf{q}'} \Gamma_{\alpha,\mathbf{q};\alpha,\mathbf{q}'} (f^{2\mathrm{D},\circ}_\alpha(\mathbf{r}, \mathbf{q}', t) - f^{2\mathrm{D}}_\alpha(\mathbf{r}, \mathbf{q}', t)) \approx 0$, where $\Gamma_{\alpha,\mathbf{q};\alpha,\mathbf{q}'}$ are the scattering

coefficients from exciton $|\alpha, \mathbf{q}'\rangle$ into $|\alpha, \mathbf{q}\rangle$ [26]. Such scattering coefficients provide the state-dependent scattering times $\tau^{-1}_{\alpha,\mathbf{q}} = \sum_{\mathbf{q}'} \Gamma_{\alpha,\mathbf{q}';\alpha,\mathbf{q}}$ [26]. Here, we assume a state-independent $\tau_{\alpha,\mathbf{q}} = \tau_p$ with the microscopically obtained phonon-driven scattering rate τ_p provided in Ref. [27] for $MoSe_2$.

While the first two terms in Eq. (2) are present also in regular TMD monolayers, the third and the fourth term are specific to lateral TMD heterostructures. The third term describes the drift of excitons driven by the energy offset at the interface and reads

$$\dot{f}^{\rm 2D,d}_{\alpha}(\mathbf{r}, \mathbf{q}, t) = -\hbar^2 \int d\mathbf{q}' \mathcal{V}_{\rm 2D}(\mathbf{r}, \mathbf{q} - \mathbf{q}') f^{\rm 2D}_{\alpha}(\mathbf{r}, \mathbf{q}', t) = -\hbar \int d\mathbf{q}' \delta(q_y - q'_y) \mathcal{V}_{\rm 1D}(x, q - q') f^{\rm 2D}_{\alpha}(\mathbf{r}, \mathbf{q}', t). \tag{4}$$

Here, $\mathcal{V}$ is induced by the excitonic energy offset and obtained by introducing the interface excitonic potential $E_\alpha(x) = E_{\rm Mo} + (E_{\rm W} - E_{\rm Mo})(1 - \tanh(4x/w))/2$, cf. Sec. 1 [28]. For the specific case, it reads

$$\mathcal{V}_{\rm 2D}(\mathbf{r}, \mathbf{q}'') = \frac{i}{(2\pi)^2\hbar^3} \int d\mathbf{r}' \left[E_\alpha\left(\mathbf{r} + \frac{\mathbf{r}'}{2}\right) - E_\alpha\left(\mathbf{r} - \frac{\mathbf{r}'}{2}\right) \right] e^{-\imath \mathbf{q}''\cdot\mathbf{r}'} = \frac{\delta(q''_y)}{\hbar} \mathcal{V}(x, q''_x)\,, \tag{5a}$$

$$\text{with } \mathcal{V}_{\rm 1D}(x, q''_x) = \frac{i}{2\pi\hbar^2} \int_{-\mathrm{X}_V}^{\mathrm{X}_V} dx' \left[E_\alpha\left(x + \frac{x'}{2}\right) - E_\alpha\left(x - \frac{x'}{2}\right) \right] e^{-\imath q''_x x'} \quad , \tag{5b}$$

where we used the property of the LH interface potential to be invariant along the interface ($E_\alpha(\mathbf{r}) \equiv E_\alpha(x)$) and introduced a reasonable cutoff of X_V =25 nm. Taylor expanding $E_\alpha\left(x \pm \frac{x'}{2}\right)$ around x and after using $x'^n e^{-\imath q'' x'} = (-1/\imath)^n \partial^n / \partial q''^n e^{-\imath q'' x'}$ together with a partial integration, one can show that [26]

$$\dot{f}^{\rm 2D,\,d}_{\alpha}(\mathbf{r}, \mathbf{q}, t) \approx \sum_n c_n \nabla^n_{\mathbf{r}} E_\alpha(\mathbf{r}) \cdot \nabla^n_{\mathbf{q}} f^{\rm 2D}_{\alpha}(\mathbf{r}, \mathbf{q}, t) \tag{6}$$

with complex constants c_n. For smooth-enough energies $E_\alpha(\mathbf{r})$, the first order of the expansion is enough, leading to the well-known semiclassical drift $\dot{f}^{\rm 2D,d}_{\alpha} = \nabla_{\mathbf{r}} E_\alpha(\mathbf{r}) \cdot \nabla_{\mathbf{q}} f^{\rm 2D,d}_{\alpha}/\hbar$. However, for realistic interface energies $E_\alpha(x)$ varying within a width $w = 2.4$ nm, all terms in Eq. (6) should be included.

Finally, the last term in Eq. (2) describes the trapping of $MoSe_2$ and WSe_2 excitons into the bound CT states. Similarly to the case of carrier-capture [29–31], this is expected to be driven by scattering with phonons and be local, i.e. taking place only when $MoSe_2/WSe_2$ excitons are located at the interface, where CT excitons are localized. In analogy to the intraband phonon-driven dynamics (second term in Eq. (2)), this is expressed in the relaxation time approximation as

$$\dot{f}^{\rm 2D,cap}_{\alpha} = \frac{\Delta n_\alpha f^{\rm 2D,\circ}_{\alpha} - f^{\rm 2D}_{\alpha}}{\tau_c}. \tag{7}$$

Here, we have introduced $\Delta n_\alpha = \frac{n^\circ_\alpha(\mathbf{r},t)}{n_\alpha(\mathbf{r},t)}$ as the ratio between the spatiotemporal exciton density $n_\alpha(\mathbf{r}, t)$ and its thermalized density $n^\circ_\alpha(\mathbf{r}, t)$. This is obtained after the introduction of CT excitons in the local thermalized spatial density of α =Mo, W excitons as $n^\circ_\alpha(\mathbf{r}, t) = d_\alpha(x)\,(n_\alpha(\mathbf{r}, t) + n_{\rm CT}(\mathbf{r}, t))$ with

$$d_\alpha(x) = \left(1 + e^{-\frac{E_{\rm CT} - E_\alpha(x)}{k_B T}} |\psi_{\rm CT}(x)|^2 \sqrt{\frac{2\pi\hbar^2}{M k_B T}} \sum_{q_y} e^{-\frac{\hbar^2 q_y^2}{2M k_B T}} \right)^{-1} . \tag{8}$$

Since the capture is dominated by the emission of optical phonons, we assume $\tau_c = \tau_{{\rm abs},c} e^{-\Omega/k_B T} / \left(1 + e^{-\Omega/k_B T}\right)$, where $\tau_{{\rm abs},c}$ is the temperature-dependent scattering time of $MoSe_2$ due to absorption of intravalley optical phonons with an energy of $\Omega = 30$ meV, which has been microscopically evaluated in Ref. [27]. Equation (7) implies a decrease of $n_\alpha(\mathbf{r}, t)$ as a consequence of trapping only when d_α differs from one. In agreement with the locality of carrier capture [29–31], this takes place only where the wavefunction $\psi_{\rm CT}(x)$ of the bound CT exciton state is finite, hence close to the interface. Furthermore, the trapping is suppressed once the ratio between n_α and $n_{\rm CT}$ approaches the equilibrium value ($n_\alpha \approx n^\circ_\alpha$). This takes place when the CT exciton occupation is high enough to have a compensation between escape and trapping. As shown in the main manuscript, this compensation takes place on very different timescales depending on the temperature of the system.

Along the interface there is no energy offset, resulting in a drift-less diffusion [32, 33], while we expect a propagation across the interface driven by the energy offset. This can be described by a one-dimensional Wigner function $f_\alpha(x, q_x, t)$

obtained by integrating over space- and momentum along the interface yielding

$$f_\alpha(x,q,t) = \frac{1}{L_y}\sum_{q_y}\int dy f_\alpha^{2\mathrm{D}}(\mathbf{r}_{CM},\mathbf{q}_{CM},t) \tag{9}$$

and for its temporal evolution

$$\dot{f}_\alpha(x,q_x,t) = \frac{1}{L_y}\sum_{q_y}\int dy \dot{f}_\alpha^{2\mathrm{D}}(\mathbf{r},\mathbf{q},t) = -v_{q_x}\frac{\partial}{\partial_x}f_\alpha + \frac{f_\alpha^\circ - f_\alpha}{\tau_p} + \dot{f}_\alpha^{\mathrm{d}} + \dot{f}_\alpha^{\mathrm{cap}} \tag{10a}$$

$$\text{with} \quad \dot{f}_\alpha^{\mathrm{d}}(x,q_x,t) = \sum_{q'_x}\mathcal{V}(x,q_x-q'_x)f_\alpha(x,q'_x,t) \quad \text{and} \quad \dot{f}_\alpha^{\mathrm{cap}} = \frac{\Delta n_\alpha f_\alpha^\circ - f_\alpha}{\tau_c} \quad . \tag{10b}$$

To obtain Eq. (10) we have assumed an identical distribution at the edges, i.e. $f_\alpha^{2\mathrm{D}}\left((x,L_y/2),\mathbf{q},t\right) \approx f_\alpha^{2\mathrm{D}}\left((x,-L_y/2),\mathbf{q},t\right)$ and introduced $f_\alpha^\circ(x,q,t) = \frac{1}{L_y}\sum_{q_y}\int dy f_\alpha^{2\mathrm{D}\circ}(\mathbf{r}_{CM},\mathbf{q}_{CM},t)$ and $\mathcal{V}(x,q_x) = -2\pi\hbar/L_x\mathcal{V}_{1\mathrm{D}}(x,q_x)$. The trapping of $MoSe_2$ and WSe_2 excitons forms CT excitons, whose spatial density $n_{\mathrm{CT}}(x,t)$ has a profile across the interface determined by the wavefunction $\psi_{\mathrm{CT}}(x)$, hence $n_{\mathrm{CT}}(x,t) = |\psi_{\mathrm{CT}}(x)|^2 N_{\mathrm{CT}}(t)$, where $N_{\mathrm{CT}}(t)$ is the time-dependent total population of CT excitons. The shape of the spatial density $n_{\mathrm{CT}}(x,t)$ stems from the general definition [23, 28] of the spatial density $n_{\mathrm{CT}}(\mathbf{r}) = \sum_{\beta\beta'}\langle\hat{X}_\beta^\dagger\hat{X}_{\beta'}\rangle\Psi_\beta(\mathbf{r})\Psi_{\beta'}^\dagger(\mathbf{r})$ for the center-of-mass wavefunction $\Psi_\beta(\mathbf{r}) \equiv \Psi_{n,q_y}(\mathbf{r}) = \psi_{n,1s}(x)\frac{1}{\sqrt{L_y}}e^{\imath q_y y}$ after focusing on the relevant ground state $n = n' = 1$. As a consequence, Eq. (10) is coupled with the dynamics of the CT exciton density by

$$\dot{n}_{\mathrm{CT}}(x,t) = |\psi_{\mathrm{CT}}(x)|^2\,\dot{N}_{\mathrm{CT}}(t), \tag{11}$$

with the temporal evolution of $N_{\mathrm{CT}}(t)$ following from the particle conservation as

$$\dot{N}_{\mathrm{CT}}(t) = -\frac{1}{L_x}\sum_{q_x}\sum_{\alpha=\mathrm{Mo,W}}\int dx \dot{f}_\alpha^{\mathrm{cap}}(x,q_x,t). \tag{12}$$

3. PHOTOLUMINESCENCE

The optical excitation of TMD-based heterostructure leads to a coherent-to-incoherent population transfer [24, 34] followed by an energy-relaxation [24, 35, 36] potentially relevant also for transient transport phenomena [23, 37]. However, such transient effects last only few hundreds of femtoseconds at high temperatures [23], a timescale much shorter than the one investigated here. As a consequence, we start from an initial exciton distribution in the form of

$$f_\alpha^\circ(x,q_x,t=0) = L_x\bar{f}_\alpha^\circ(q_x)e^{-\frac{(x-x_L)^2}{2\Delta_0^2}}\,g_0(x) \quad , \tag{13}$$

where $\bar{f}_\alpha^\circ(q_X) = 1/L_x\frac{\sqrt{2\pi}\hbar}{\sqrt{Mk_BT}}\exp\left[-\frac{\hbar^2q_X^2}{2Mk_BT}\right]$ is the normalized Boltzmann distribution, while X_L and Δ_0 provide the position and the size of the laser spot, respectively. Finally, g_0 provides spatial inhomogeneities induced by the energy selection rule: in the case of an excitation resonant to WSe_2 we set $g_0(x) = \Theta(-x) + c_L\Theta(x)$, with $c_L = 0.01$ leading to a much larger excitation at the WSe_2 side ($x < 0$). In view of the 90 meV separation between the laser energy and E_{Mo}, the $MoSe_2$ excitons are formed less efficiently via phonon-driven non-resonant processes [38, 39]. In contrast, for the case of non-resonant excitations we expect only minor differences in the formation of WSe_2 and $MoSe_2$ excitons, leading to $g_0 = 1$.

The solution of Eq. (1) provides exciton wavefunctions, which allows to determine the oscillator strength γ_α. The latter is proportional to the probability $|\phi(\mathbf{r}=0)|^2 = \int dR_x|\psi(R_x)|^2|\phi^{R_x}(\mathbf{r}=0)|^2$ of finding electrons and holes in the same position [1, 2]. In the specific case of the hBN-encapsulated WSe_2-$MoSe_2$ lateral heterostructure, the spatial separation of CT excitons leads to an oscillator strength that is about 35 times smaller than for $MoSe_2$ or WSe_2 excitons [2]. The spatial dipole furthermore leads to smaller binding energies is analogy to the case of interlayer excitons in vertical heterostructures [2, 40]. The energy-, space- and time-resolved photoluminescence $I(E,x,t)$ (PL) is determined by a product of the exciton density and the oscillator strength of the involved exciton species resulting

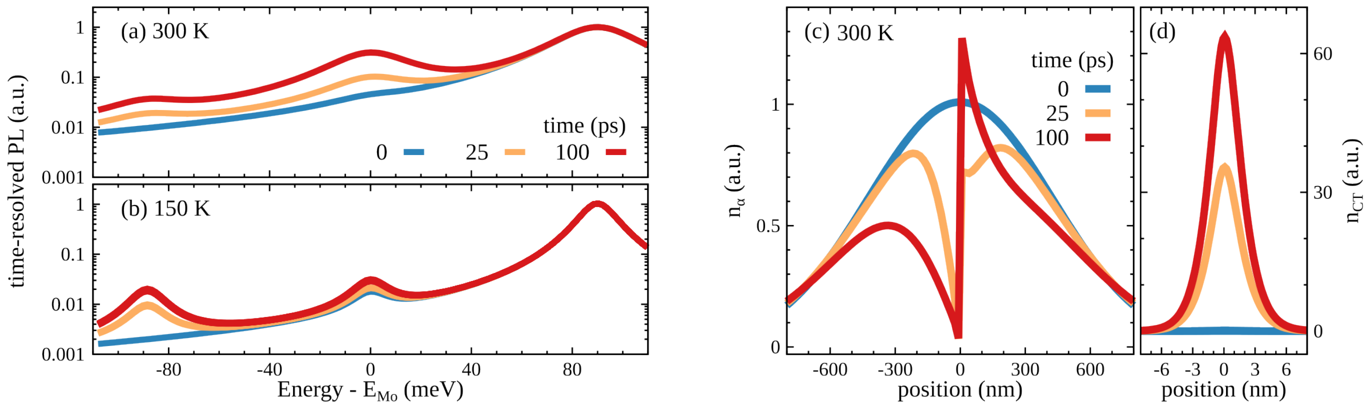

Fig. S 2: (a-b)Energy- and time-resolved photoluminescence (PL) spectrum normalized to the X_W peak after micrometer excitation at the interface in resonance with X_W at (a) 300K and (b) 150 K. At 300 K, the X_{Mo} resonance becomes comparable to X_W after approximately 100 ps reflecting the efficient exciton drift across the interface. Such a propagation is suppressed at the smaller temperature of 150 K, resulting in X_W remaining much larger than X_{Mo}. Furthermore, a small X_{CT} peak emitted from CT excitons appear after few hundreds of picoseconds. (c-d) Space-resolved densities of (a) WSe_2 and $MoSe_2$ excitons as well as of (b) CT excitons at 300 K after an non-resonant optical excitation at the interface. Similarly to the case of a resonant excitation (Fig. 2 of the main manuscript) we find the appearance of exciton depletion and accumulation at the WSe_2 and $MoSe_2$ side of the interface, respectively. At the same time, we observe the build-up of a large CT-exciton density that is about two orders of magnitude larger than the optically excited density.

in an Elliot formula [41, 42], which we have generalized to include CT excitons [2]

$$I(E, x, t) = \sum_{\alpha} \frac{f_{\alpha}^{2D,\circ}(x, \mathbf{q} = 0, t)\, \tilde{\gamma}_{\alpha}\, (\tilde{\gamma}_{\alpha} + \Gamma)}{(E - E_{\alpha})^2 + (\tilde{\gamma}_{\alpha} + \Gamma)^2}. \tag{14}$$

The PL is influenced by the temperature-dependent exciton-phonon scattering rate $\Gamma = \hbar/2\tau_p$ and the radiative decay rate $\tilde{\gamma} = \gamma_{\alpha}|\psi_{\alpha,\mathbf{q}=0}|^2$, which is proportional to the $q = 0$ component of the squared wavefunction in center-of-mass momentum ψ_{q_x}. This is due to the conservation of momentum during the emission process [43]. Importantly, the oscillator strength for CT excitons γ_{CT} is 35 smaller than for the intralayer one $\gamma_{Mo/W}$ due to the large spatial separation between electrons and holes, which inhibits their recombination [2]. Finally, for the case of the time-integrated PL $\bar{I}(E, x) = \int dt I(E, x, t)e^{-t/\tau}$ we consider an exciton lifetime τ =175 ps according to recent experiments in LHs [13].

In the main manuscript (Fig. 3(c)) we showed the time-resolved evolution of the energy- and time-resolved, space-integrated PL after resonant excitation at the interface at room temperature. In Fig. S2(b) we illustrate the PL at 150 K. For a direct comparison, in Fig. S2(a) we show again the results at 300 K, now taking cuts at given times. In this way, we clearly observe the delayed appearance of the X_{Mo} resonance, which is formed due to exciton propagation across the interface driven by the energy offset of the optically excited WSe_2 excitons. Thanks to this unidirectional propagation, the X_{Mo} resonance becomes as intense as X_W already after few hundreds of picoseconds. Furthermore, the time-resolved PL allows to resolve a small peak X_{CT} emitted by CT excitons. The situation becomes significantly different at 150 K, cf. Fig. S2(b). Here, the trapping into CT excitons becomes so efficient that only few optically excited WSe_2 excitons are able to cross the interface. As a consequence, the X_{Mo} resonance remains one order of magnitude smaller than the X_W peak even after 150 ps. The height of X_{Mo} increases only weakly compared to its initial value induced by the residual non-resonant excitation ($c_L = 0.01$ in Eq. (13)). Furthermore, contrary to the room temperature case also the X_{CT} resonance becomes as intense as X_{Mo}. While the transport across the interface is suppressed by the decreasing temperature, the trapping still takes place, resulting in the formation of the X_{CT} resonance.

In the main manuscript, we have also considered a far-field excitation (laser spot of 1 μm) resonant to the WSe_2 exciton energy. In Fig. S2(c-d), we show the same situation, but now after a high-energy excitation, resulting in an initial exciton density being spatially symmetric around the interface, cf. the cyan line in Fig. S2(c). Exploring the spatiotemporal exciton dynamics, we observe (i) an accumulation of exciton density at the $MoSe_2$ side and (ii) a depletion area at the WSe_2 side of the interface, cf. Fig. S2(c). This is induced by the unidirectional propagation driven by the energy offset between the two sides. Furthermore, we find the formation of (iii) a large CT exciton density, cf. Fig. S2(d). It becomes one (two) orders of magnitude larger than the initial exciton density after approximately 10 ps (100 ps).

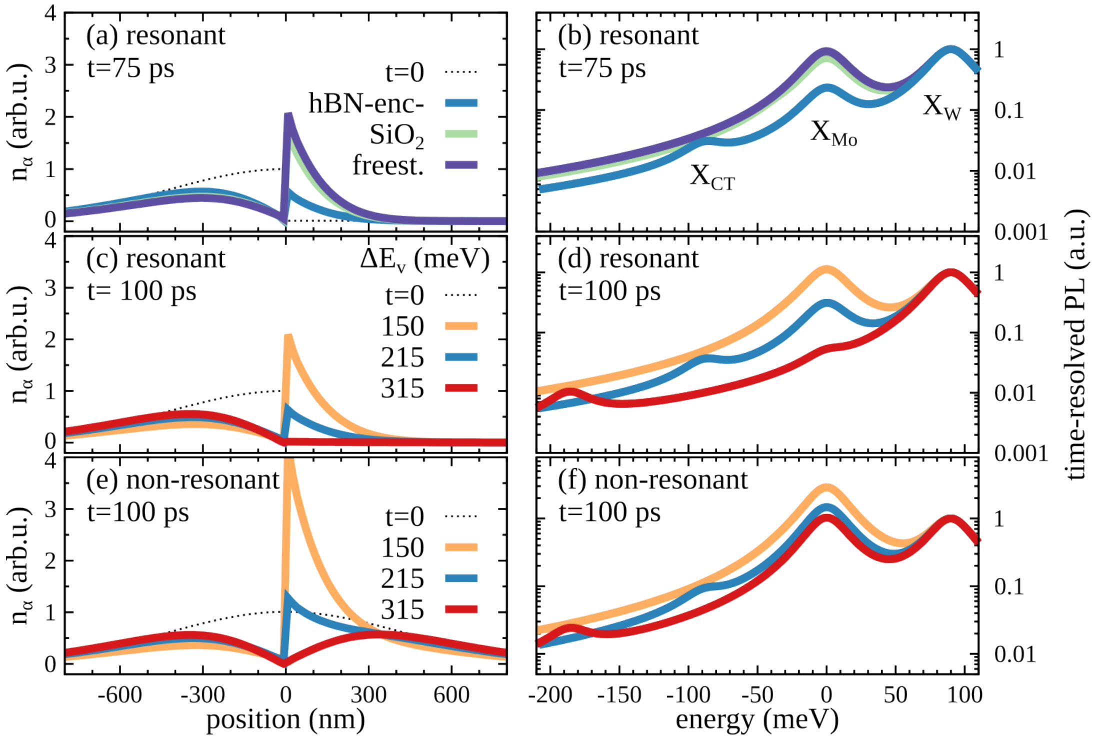

Fig. S3: **Interface and dielectric engineering.** (a) Spatial profile of the WSe_2 and $MoSe_2$ exciton density at 75 ps after an excitation at the interface of a lateral WSe_2-$MoSe_2$ heterostructure for different dielectric environments and resonant to the energy of WSe_2 excitons. (b) The corresponding PL spectrum normalized to the peak of WSe_2 excitons. Samples deposited on SiO_2 or free-standing samples exhibit a smaller impact of CT excitons, resulting in a higher accumulation of excitons at the $MoSe_2$ side of the interface and thus in a higher X_{Mo} peak. (c) Spatial profile of the exciton density in hBN-encapsulated lateral TMD heterostructures at a fixed time of 100 ps and with different valence-band energy offsets ΔE_v with (d) the corresponding PL spectrum. There is a minimum ΔE_v required to observe CT excitons in the PL. (e,f) Same as in (c,d), but now considering a non-resonant excitation.

4. DIELECTRIC- AND INTERFACE ENGINEERING OF EXCITON TRANSPORT

In the main manuscript, we have discussed how temperature can be used as an externally accessible tuning knob for unidirectional exciton transport and exciton capture at the interface of lateral TMD heterostructures. Now, we investigate several other knobs including dielectric and interface engineering (variation of the interface width, alloying, and energetic band offsets). These allow controlling the impact of CT excitons by modifying their energy relative to monolayer excitons [2]. Substrates with a small dielectric constant reduce the energy separation $E_{\mathrm{Mo}} - E_{\mathrm{CT}}$ between monolayer and CT excitons. We find $E_{\mathrm{Mo}} - E_{\mathrm{CT}} \approx 88, 35$ and 6 meV for hBN-encapsulated samples, samples deposited on SiO_2 and free-standing samples, respectively. Smaller energy separations result in a weaker trapping into CT excitons, hence inducing a more efficient unidirectional exciton transport across the interface. To show this, in Fig. S3(a) we consider the spatial exciton distribution after a resonant excitation, similar to Figs. 1 and 2 of the main manuscript. At 75 ps, we find the highest accumulation of excitons on the $MoSe_2$ side in the free-standing case, i.e., in the dielectric environment which minimizes the impact of CT excitons. Exciton accumulation is accessible in spatially resolved experiments, but the unidirectional drift and its dependence on the dielectric environment can also be measured in space-integrated PL experiments via the change of PL intensity of the peak ratio between X_{Mo} and X_{W} excitons, cf. Fig. S3(b). The more efficient the unidirectional transport is, the higher is the $X_{\mathrm{Mo}}/X_{\mathrm{W}}$ ratio. We also reveal the emergence of a small X_{CT} peak stemming from CT excitons, which is absent for smaller dielectric constants due to the weaker exciton trapping at the interface.

While the unidirectional exciton drift is governed by different energy bandgaps at the two sides of the interface, the single-particle offset ΔE_v in the valence bands determines the energy separation $E_{\mathrm{Mo}} - E_{\mathrm{CT}}$ [2]: the smaller ΔE_v, the less important CT excitons are. The resulting impact on exciton transport leads to an increased accumulation of excitons at the $MoSe_2$ side for smaller ΔE_v, as the capture process at the interface becomes negligible, cf. Fig. S3(c). As a result, there is an increased PL peak stemming from X_{Mo} excitons for smaller offsets ΔE_v, cf. Fig. S3(d). Moreover, for increasing ΔE_v we find the emergence of a low-energy X_{CT} peak. The height of this peak depends on the

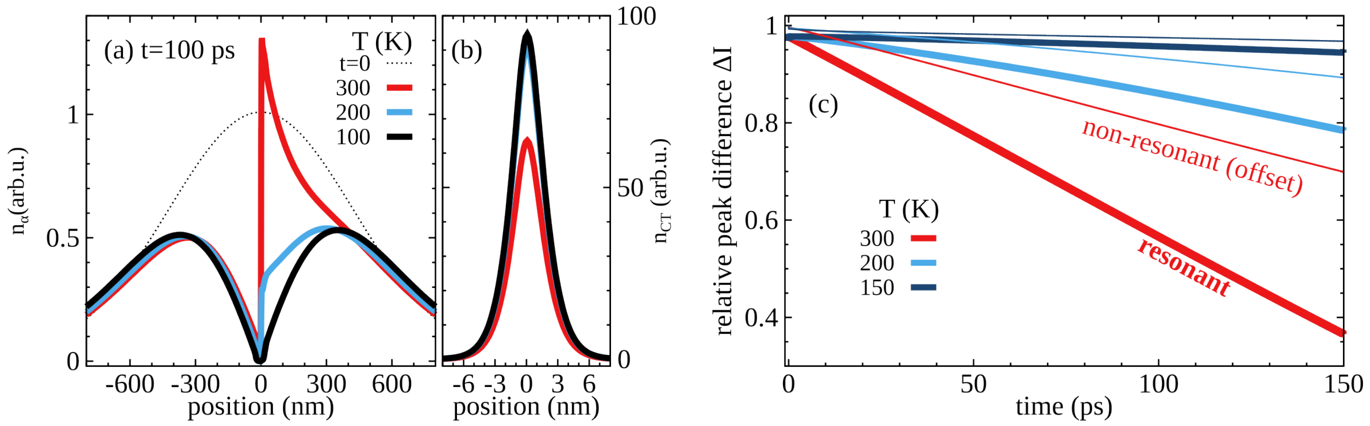

Fig. S 4: **Cryogenic exciton transport.** Spatial profile of (a) WSe_2 and $MoSe_2$ as well as of (b) CT excitons at 100 ps after an excitation at the interface of a hBN-encapsulated lateral WSe_2-$MoSe_2$ heterostructure for different temperatures. The drift-induced accumulation of excitons at the $MoSe_2$ side of the interface can be observed only for moderate/high temperatures. (c) Evolution of the relative peak difference $\Delta I = \frac{I_W - I_{Mo}}{I_W + I_{Mo}}$ between WSe_2 and $MoSe_2$ excitons. The predicted decrease of ΔI in time reveals the unidirectional transport of optically excited WSe_2 excitons across the interface, which is more efficient at high temperatures and resonant excitation.

exciton oscillator strength. Considering an offset ΔE_v=215 meV present in hBN-encapsulated lateral WSe_2-$MoSe_2$ heterostructures, we predict a CT-exciton oscillator strength that is 35 times smaller than for the monolayer states [2]. Increasing the offset to 315 meV, the oscillator strength further decreases by more than a factor of 3, hence becoming roughly two orders of magnitude smaller than monolayer excitons. This can be traced back to the reduction of the binding energy of CT excitons [2].

The unidirectional exciton transport across the interface and the resulting spatial accumulation of excitons are robust with respect to different excitation conditions. In Figures S3(e)-(f) we show the spatial distribution of excitons and the corresponding PL spectrum for varying ΔE_v in the case of an initial equal occupation of the WSe_2 and $MoSe_2$ excitons, corresponding to a non-resonant excitation half way between X_{Mo} and X_W excitons. In this excitation scheme, the drift-induced accumulation builds on top of a large initial $MoSe_2$ occupation, resulting in a higher density compared to the resonant case, cf. Figs. S3(c) and (e). On the other hand, the PL spectrum on logarithmic scale presents quantitatively smaller changes upon energy offset variations, cf. Figs. S3(d) and (f). Both features reflect the higher initial occupation of $MoSe_2$. Importantly, all qualitative statements obtained for the resonant excitation are valid also for the non-resonant case, in particular the predicted decrease in exciton drift for increasing energy offsets (Fig. S3(e)) or decreasing temperatures.

In the main manuscript, we have shown how the suppression of exciton transport across the interface can be observed in the intermediate/high temperature regime. Already at 150K, the unidirectional transport is essentially suppressed, as reflected by the negligible accumulation of excitons at the $MoSe_2$ side of the interface (and the small spatial offset $\Delta_X \approx$ 20nm in near-field experiments (Figs. 3 and 4 in the main manuscript, respectively). Here, we extend the range of temperatures down to 100 K, leading to a drastic reduction of exciton transport resulting basically in no exciton accumulation, cf. Fig. S4(a). The formation of a dip at the interface reveals the presence of an efficient trapping into CT excitons (cf. Fig. S4(b)). The latter can only be compensated for at higher temperatures by an efficient exciton drift across the interface resulting in an accumulation of excitons at the energetically favorable side of the interface.

Besides temperature, excitation conditions also allows controlling the response of lateral heterostructures. Concerning the impact of a non-resonant excitation of $MoSe_2$ excitons, Figs. S3(c),(e) as well as Fig. 3 of the main manuscript and Fig. S4(a) compare directly the resonant and the non-resonant case. In general, the comparison indicates that the qualitative trend of the unidirectional exciton transport across the interface remains the same, independently of the excitation energy. To be able to quantify this, in Fig. S4(c) we directly monitor the evolution of the relative peak difference of X_W and X_{Mo} excitons, i.e.

$$\Delta I = \frac{I_W - I_{Mo}}{I_W + I_{Mo}} \quad , \qquad (15)$$

where $I_\alpha \equiv I_\alpha(t)$ provides the energy-integrated time-resolved PL around the X_α peak. Assuming a similar intensity per exciton from the two sides of the interface as found in recent experiments [13], this quantity corresponds to the relative total occupation difference $\Delta I \approx \Delta N$, with $\Delta N = \frac{N_W - N_{Mo}}{N_W + N_{Mo}}$ and $N_\alpha \equiv N_\alpha(t) = \int dx n_\alpha(x,t)$ being

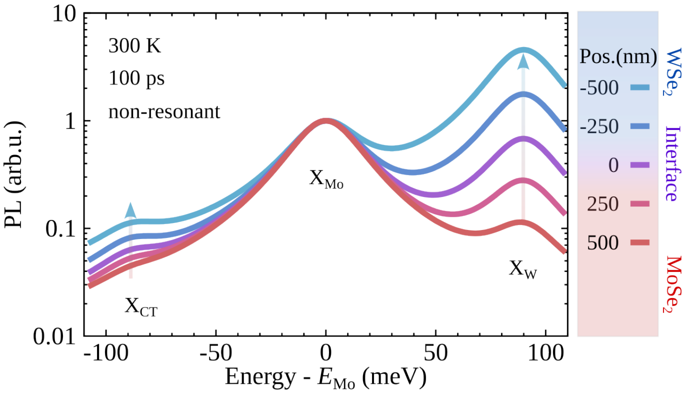

Fig. S5: **Far-field laser spectroscopy.** Time-resolved space-integrated PL spectrum for far-field non-resonant excitations as a function of laser position. Exciting at the WSe_2 side of the lateral heterojunction not only leads to the increase of X_W, but also to an increased intensity of X_{CT} relative to X_{Mo}.

the spatially integrated occupation of excitons α=Mo,W. In the case of a resonant excitation, one has $\Delta I \approx 1$ at t=0, with a small deviation from 1 induced by the residual initial occupation $N_{Mo} \approx 0.01 N_W$. As time passes by, ΔI decreases due to the transport-induced formation of $MoSe_2$ excitons. The speed of such a process depends drastically on temperature: At 300 K, ΔI approaches 0 after 250 ps, indicating that $MoSe_2$ and WSe_2 excitons are equally occupied. In contrast, at 100 K the relative peak difference remains almost unchanged, indicating a strongly suppressed transport across the interface, as WSe_2 excitons become trapped at the interface before reaching the $MoSe_2$ side. Considering now a non-resonant excitation, we find that the decrease of ΔI is roughly two times slower than in the resonant case.

For near-field excitations, we have already shown a study varying the position of the laser spot, cf. Fig. 4 of the main manuscript. The position of the laser spot can lead to interesting results also for far-field experiments, as we show in Fig. S5. As expected, moving the laser spot into the WSe_2 side leads to an increased intensity of X_W relative to X_{Mo}. Interestingly, exciting into WSe_2 also results in an increased intensity of X_{CT} relative to X_{Mo}, in agreement with previous experiments and predictions [2, 13].

Now, we vary the laser spot size Δ_0 corresponding to the spatial FWHM of the Guassian divided by a factor of $2\sqrt{2\ln 2}$, cf. Eq. (13). In Fig. S6(a,b) we explore the evolution of the corresponding ΔI, which is characterized by a clear decrease as a function of time. For smaller Δ_0, the decrease is much faster. Comparing the resonant and the non-resonant case, we find that the decay in the first case is always roughly two times faster, cf. Figs. S6(a) and S6(b). This can be directly quantified in Fig. S6(c), where we investigate the average decrease $\langle \partial_t \Delta I \rangle_{t_0}$ of ΔI in the first ps, i.e. $\langle \partial_t \Delta I \rangle_{t_0} = \frac{1}{t_0} \int dt \partial_t \Delta I$ with $t_0 = 1$ps. Such an averaging minimizes transient effects induced by the ultrafast sub-picosecond dynamics following the instantaneous generation of the exciton density (cf. Eq. (13)), after which ΔI evolves almost linearly with time, cf. Figs. S6(a,b). In Fig. S6(c) we show how the averaged variation $\langle \partial_t \Delta I \rangle_{t_0}$ becomes more effective for smaller Δ_0, revealing also the already observed factor of two difference between the resonant and non-resonant case.

To understand this behaviour, we introduce a simplified drift-diffusion equation to describe the spatiotemporal exciton dynamics

$$\partial_t n_\alpha(x,t) = -v_{\text{eff}}(t)\partial_x n_\alpha(x,t) + D\partial_x^2 n_\alpha(x,t) - \frac{n_\alpha(x,t)}{\tau} \quad , \tag{16}$$

where the second term provides the diffusion while the third contains all decaying mechanisms (including both capture into CT excitons and overall decay due to other radiative and non-radiative processes). Importantly, the first term include the drift, which is now quantified by an effective velocity $v_{\text{eff}}(t)$. Starting from the results of our full simulations obtained from Eqs. (10), we can use the evolution of $\langle \partial_t \Delta I \rangle_{1ps}$ to estimate the effective velocity $v_{\text{eff}}(t)$ of the unidirectional exciton transport. To do so, we spatially integrate Eq. (16) to get the evolution of the total WSe_2 exciton population $N_W(t) = \int dx\, n_W(x,t)$ as

$$\partial_t N_W = \int_{-\infty}^{0} dx \partial_t n_W(x,t) \quad . \tag{17}$$

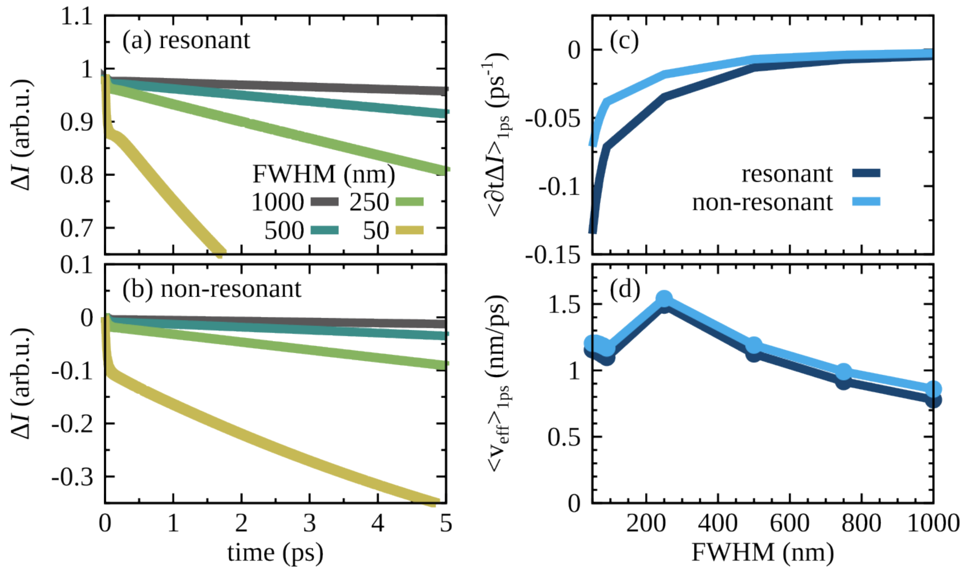

Fig. S6: **Relative peak difference and effective drift velocity.** Time-resolved evolution of the relative peak difference ΔI for different spot sizes and (a) resonant and (b) non-resonant excitation. This shows a steeper variation of ΔI with a decreasing initial localization and with resonant excitation, as revealed by (c) its time-derivative averaged over the first picosecond. (d) The resulting effective drift velocity v_{eff} is robust against excitation scheme, with values of about 1 nm/ps.

In the early phase of the evolution, the shape of $n_{\mathrm{W}}(x,t)$ can still be approximated by a Gaussian as in Eq. (13). Further assuming that initially the impact of the decay is smaller compared to the drift, $\frac{n_\alpha(x,t)}{\tau} \ll -v_{\mathrm{eff}}(t)\partial_x n_\alpha(x,t)$, and using $\partial_x n_{\mathrm{W}}(x=0,-\infty) \approx 0$ we get

$$\partial_t N_{\mathrm{W}}(t) \approx -v_{\mathrm{eff}}(t) n_{\mathrm{W}}(x=0,t) \approx -v_{\mathrm{eff}}(t)\sqrt{\frac{2}{\pi}}\frac{1}{\Delta_0}N_{\mathrm{W}}(t) \quad . \tag{18}$$

On the other hand, assuming again $\Delta I \approx \Delta N$ we find

$$\partial_t \Delta I(t) \approx \partial_t \Delta N(t) \approx \frac{1}{N_{\mathrm{W}}(t)+N_{\mathrm{Mo}}(t)} 2\partial_t N_{\mathrm{W}}(t) \quad , \tag{19}$$

where we used that in the limit of a weak decay $N_{\mathrm{W}} + N_{\mathrm{Mo}}$ is constant and $\partial_t N_{\mathrm{W}} = -\partial_t N_{\mathrm{Mo}}$ with $\partial_t N_{\mathrm{Mo}}$ being the straight-forward generalization of Eq. (18). Finally, using $N_{\mathrm{W}}/(N_{\mathrm{W}}+N_{\mathrm{Mo}}) \approx I_{\mathrm{W}}/(I_{\mathrm{W}}+I_{\mathrm{Mo}})$ from Eqs. (18) and (19), we find

$$\partial_t \Delta I(t) \approx -v_{\mathrm{eff}}\sqrt{\frac{2}{\pi}}\frac{1}{\Delta_0}\frac{I_{\mathrm{W}}}{I_{\mathrm{W}}+I_{\mathrm{Mo}}} \quad . \tag{20}$$

For a given v_{eff}, Eq. (20) implies that the decrease of $\Delta I(t)$ with time i) scales with $1/\Delta_0$ and ii) is twice larger for the resonant case, as the ratio $\frac{I_{\mathrm{W}}}{I_{\mathrm{W}}+I_{\mathrm{Mo}}}$ approaches the values of 1 and 1/2 in the resonant and the non-resonant case. This behaviour is in perfect agreement with the results of Fig. S7, obtained without approximations described above. Moreover, the simplified equation allows to define and effective dirft velocity

$$v_{\mathrm{eff}}(t) = \Delta_0\sqrt{\frac{\pi}{2}}\left(1+\frac{I_{\mathrm{Mo}}}{I_{\mathrm{W}}}\right)\partial_t \Delta I(t) \quad . \tag{21}$$

The resulting value of $\langle v_{\mathrm{eff}}\rangle_{1ps}$ is plotted in Fig. S7(d). Interestingly, we see that the value is extremely robust upon variation of both spot size and excitation energy. We only see a slight increase for Δ_0 corresponding to a FWHM of 250 nm, which could stem from the competition between diffusion and drift [44, 45].

The non-monotonic variation of Δ_X with temperature in Fig. 4(c) of the main manuscript is induced by the competition of the energy-offset-induced exciton drift and the trapping/excape into/from CT excitons at the interface. Remarkably, these two effects show an opposite behaviour with respect to temperature. To better illustrate this, in Fig. S7 we consider the temperature-dependent spatial offset Δ_X. We compare the full simulation with the

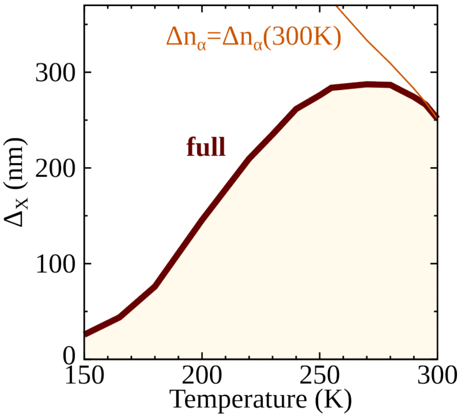

Fig. S7: **Spatial offset.** Temperature-dependent spatial offset Δ_X (as defined in Fig. 4 of the main manuscript) comparing the full simulation with the gedanken experiments, where the trapping is made temperature-independent by assuming a thermalized exciton ratio $\Delta n_\alpha(T) \equiv \Delta n_\alpha(300K)$. In this gedanken case, Δ_X increases with decreasing temperatures due to the increase of mobility and ratio between equilibrium distribution of $MoSe_2$ and WSe_2 excitons.

artifical case, where the temperature activation of trapping is removed by assuming a thermalized exciton ratio $\Delta n_\alpha(T) \equiv \Delta n_\alpha(300K)$. The latter provides the ratio between the spatiotemporal exciton density $n_\alpha(x,t)$ and the thermalized density $n_\alpha^\circ(x,t)$ after introduction of CT excitons (for α=W,Mo). This ratio affects the capture into CT excitons, cf. Eqs. (7) and (8). In this artificial case, taking a constant ratio $\Delta n_\alpha(T)$ leads to a temperature-independent capture and escape into/from CT excitons, leading to a monotonic decrease of Δ_X for temperatures approaching 300 K. On the other hand, the realistic increase of trapping with decreasing temperatures leads to a smaller spatial offsets Δ_X. The competition between the opposite temperature-dependent variations of exciton transport induced by drift and capture leads to the predicted non-monotonic behaviour of Δ_X.

REFERENCES

[1] K. W. Lau, Calvin, Z. Gong, H. Yu, and W. Yao, Phys. Rev. B **98**, 115427 (2018), URL `https://link.aps.org/doi/10.1103/PhysRevB.98.115427`.

[2] R. Rosati, I. Paradisanos, L. Huang, Z. Gan, A. George, K. Watanabe, T. Taniguchi, L. Lombez, P. Renucci, A. Turchanin, et al., Nat. Commun. **14**, 2438 (2023), ISSN 2041-1723, URL `https://doi.org/10.1038/s41467-023-37889-9`.

[3] X. Duan, C. Wang, J. C. Shaw, R. Cheng, Y. Chen, H. Li, X. Wu, Y. Tang, Q. Zhang, A. Pan, et al., Nat. Nanotechnol. **9**, 1024 (2014), ISSN 1748-3395, URL `https://doi.org/10.1038/nnano.2014.222`.

[4] C. Huang, S. Wu, A. M. Sanchez, J. J. P. Peters, R. Beanland, J. S. Ross, P. Rivera, W. Yao, D. H. Cobden, and X. Xu, Nat. Mater. **13**, 1096 (2014), ISSN 1476-4660, URL `https://doi.org/10.1038/nmat4064`.

[5] C. Zhang, M.-Y. Li, J. Tersoff, Y. Han, Y. Su, L.-J. Li, D. A. Muller, and C.-K. Shih, Nat. Nanotech. **13**, 152 (2018), ISSN 1748-3395, URL `https://doi.org/10.1038/s41565-017-0022-x`.

[6] S. Xie, L. Tu, Y. Han, L. Huang, K. Kang, K. U. Lao, P. Poddar, C. Park, D. A. Muller, R. A. DiStasio, et al., Science **359**, 1131 (2018), URL `https://www.science.org/doi/abs/10.1126/science.aao5360`.

[7] J. Kang, S. Tongay, J. Zhou, J. Li, and J. Wu, Appl. Phys. Lett. **102**, 012111 (2013), URL `https://doi.org/10.1063/1.4774090`.

[8] Y.-H. Chu, L.-H. Wang, S.-Y. Lee, H.-J. Chen, P.-Y. Yang, C. J. Butler, L.-S. Lu, H. Yeh, W.-H. Chang, and M.-T. Lin, Appl. Phys. Lett. **113**, 241601 (2018), URL `https://doi.org/10.1063/1.5053144`.

[9] P. K. Sahoo, S. Memaran, Y. Xin, L. Balicas, and H. R. Gutiérrez, Nature **553**, 63 (2018), ISSN 1476-4687, URL `https://doi.org/10.1038/nature25155`.

[10] E. Najafidehaghani, Z. Gan, A. George, T. Lehnert, G. Q. Ngo, C. Neumann, T. Bucher, I. Staude, D. Kaiser, T. Vogl, et al., Adv. Funct. Mater. **31**, 2101086 (2021).

[11] C. Herbig, C. Zhang, F. Mujid, S. Xie, Z. Pedramrazi, J. Park, and M. F. Crommie, Nano Lett. **21**, 2363 (2021), URL `https://doi.org/10.1021/acs.nanolett.0c04204`.

[12] Y. Guo and J. Robertson, Appl. Phys. Lett. **108**, 233104 (2016).

[13] D. Beret, I. Paradisanos, H. Lamsaadi, Z. Gan, E. Najafidehaghani, A. George, T. Lehnert, J. Biskupek, U. Kaiser, S. Shree, et al., npj 2D Mater. Appl. **6**, 84 (2022), ISSN 2397-7132, URL `https://doi.org/10.1038/s41699-022-00354-0`.

[14] A. Pospischil, M. M. Furchi, and T. Mueller, Nat. Nanotechnol. **9**, 257 (2014), ISSN 1748-3395, URL `https://doi.org/10.1038/nnano.2014.14`.

[15] B. W. H. Baugher, H. O. H. Churchill, Y. Yang, and P. Jarillo-Herrero, Nat. Nanotechnol. **9**, 262 (2014), ISSN 1748-3395, URL `https://doi.org/10.1038/nnano.2014.25`.

[16] J. S. Ross, P. Klement, A. M. Jones, N. J. Ghimire, J. Yan, D. G. Mandrus, T. Taniguchi, K. Watanabe, K. Kitamura, W. Yao, et al., Nat. Nanotechnol. **9**, 268 (2014), ISSN 1748-3395, URL https://doi.org/10.1038/nnano.2014.26.

[17] D. Thureja, A. Imamoglu, T. Smoleński, I. Amelio, A. Popert, T. Chervy, X. Lu, S. Liu, K. Barmak, K. Watanabe, et al., Nature **606**, 298 (2022), ISSN 1476-4687, URL https://doi.org/10.1038/s41586-022-04634-z.

[18] M. Heithoff, Moreno, I. Torre, M. S. G. Feuer, C. M. Purser, G. M. Andolina, G. Calajò, K. Watanabe, T. Taniguchi, D. M. Kara, et al., ACS Nano **18**, 30283 (2024), pMID: 39431410, URL https://doi.org/10.1021/acsnano.4c04786.

[19] N. S. Rytova, Proc. MSU, Phys., Astron. **30**, 3 (1967).

[20] L. Keldysh, JETPL **29**, 658 (1979).

[21] S. Brem, J. Zipfel, M. Selig, A. Raja, L. Waldecker, J. D. Ziegler, T. Taniguchi, K. Watanabe, A. Chernikov, and E. Malic, Nanoscale **11**, 12381 (2019), URL http://dx.doi.org/10.1039/C9NR04211C.

[22] A. Kormányos, G. Burkard, M. Gmitra, J. Fabian, V. Zólyomi, N. D. Drummond, and V. Fal'ko, 2D Mater. **2**, 022001 (2015).

[23] R. Rosati, R. Perea-Causín, S. Brem, and E. Malic, Nanoscale **12**, 356 (2020), URL http://dx.doi.org/10.1039/C9NR07056G.

[24] S. Brem, M. Selig, G. Berghaeuser, and E. Malic, Scientific Reports **8**, 8238 (2018), ISSN 2045-2322, URL https://doi.org/10.1038/s41598-018-25906-7.

[25] J. C. König-Otto, M. Mittendorff, T. Winzer, F. Kadi, E. Malic, A. Knorr, C. Berger, W. A. de Heer, A. Pashkin, H. Schneider, et al., Phys. Rev. Lett. **117**, 087401 (2016), URL https://link.aps.org/doi/10.1103/PhysRevLett.117.087401.

[26] O. Hess and T. Kuhn, Phys. Rev. A **54**, 3347 (1996), URL https://link.aps.org/doi/10.1103/PhysRevA.54.3347.

[27] M. Selig, G. Berghäuser, A. Raja, P. Nagler, C. Schüller, T. F. Heinz, T. Korn, A. Chernikov, E. Malic, and A. Knorr, Nat. Commun. **7**, 13279 (2016), ISSN 2041-1723, URL https://doi.org/10.1038/ncomms13279.

[28] R. Rosati and F. Rossi, Phys. Rev. B **89**, 205415 (2014), URL https://link.aps.org/doi/10.1103/PhysRevB.89.205415.

[29] M. Glanemann, V. M. Axt, and T. Kuhn, Phys. Rev. B **72**, 045354 (2005), URL https://link.aps.org/doi/10.1103/PhysRevB.72.045354.

[30] D. Reiter, M. Glanemann, V. M. Axt, and T. Kuhn, Phys. Rev. B **75**, 205327 (2007), URL https://link.aps.org/doi/10.1103/PhysRevB.75.205327.

[31] R. Rosati, D. E. Reiter, and T. Kuhn, Phys. Rev. B **95**, 165302 (2017), URL https://link.aps.org/doi/10.1103/PhysRevB.95.165302.

[32] L. Yuan, B. Zheng, Q. Zhao, R. Kempt, T. Brumme, A. B. Kuc, C. Ma, S. Deng, A. Pan, and L. Huang, ACS Nano **17**, 15379 (2023), ISSN 1936-0851, URL https://doi.org/10.1021/acsnano.2c12903.

[33] X. P. Vögele, D. Schuh, W. Wegscheider, J. P. Kotthaus, and A. W. Holleitner, Phys. Rev. Lett. **103**, 126402 (2009), URL https://link.aps.org/doi/10.1103/PhysRevLett.103.126402.

[34] M. Selig, G. Berghäuser, M. Richter, R. Bratschitsch, A. Knorr, and E. Malic, 2D Mater. **5**, 035017 (2018), URL https://doi.org/10.1088%2F2053-1583%2Faabea3.

[35] R. Wallauer, R. Perea-Causin, L. Münster, S. Zajusch, S. Brem, J. Güdde, K. Tanimura, K.-Q. Lin, R. Huber, E. Malic, et al., Nano Lett. **21**, 5867 (2021), ISSN 1530-6984, URL https://doi.org/10.1021/acs.nanolett.1c01839.

[36] D. Schmitt, J. P. Bange, W. Bennecke, A. AlMutairi, G. Meneghini, K. Watanabe, T. Taniguchi, D. Steil, D. R. Luke, R. T. Weitz, et al., Nature **608**, 499 (2022), ISSN 1476-4687, URL https://doi.org/10.1038/s41586-022-04977-7.

[37] R. Rosati, K. Wagner, S. Brem, R. Perea-Causín, J. D. Ziegler, J. Zipfel, T. Taniguchi, K. Watanabe, A. Chernikov, and E. Malic, Nanoscale **13**, 19966 (2021), URL http://dx.doi.org/10.1039/D1NR06230A.

[38] S. Shree, M. Semina, C. Robert, B. Han, T. Amand, A. Balocchi, M. Manca, E. Courtade, X. Marie, T. Taniguchi, et al., Phys. Rev. B **98**, 035302 (2018), URL https://link.aps.org/doi/10.1103/PhysRevB.98.035302.

[39] C. M. Chow, H. Yu, A. M. Jones, J. R. Schaibley, M. Koehler, D. G. Mandrus, R. Merlin, W. Yao, and X. Xu, npj 2D Mater. and App. **1**, 33 (2017), ISSN 2397-7132, URL https://doi.org/10.1038/s41699-017-0035-1.

[40] S. Latini, K. T. Winther, T. Olsen, and K. S. Thygesen, Nano Lett. **17**, 938 (2017), ISSN 1530-6984.

[41] S. W. Koch, M. Kira, G. Khitrova, and H. M. Gibbs, Nat. Mater. **5**, 523 (2006), ISSN 1476-4660, URL https://doi.org/10.1038/nmat1658.

[42] S. Brem, A. Ekman, D. Christiansen, F. Katsch, M. Selig, C. Robert, X. Marie, B. Urbaszek, A. Knorr, and E. Malic, Nano Lett. **20**, 2849 (2020), ISSN 1530-6984.

[43] M. Feierabend, Z. Khatibi, G. Berghäuser, and E. Malic, Phys. Rev. B **99**, 195454 (2019), URL https://link.aps.org/doi/10.1103/PhysRevB.99.195454.

[44] M. G. Harats, J. N. Kirchhof, M. Qiao, K. Greben, and K. I. Bolotin, Nat. Photonics **14**, 324 (2020), ISSN 1749-4893, URL https://doi.org/10.1038/s41566-019-0581-5.

[45] M. G. Harats and K. I. Bolotin, 2D Mater. **8**, 015010 (2020), URL https://dx.doi.org/10.1088/2053-1583/abbabf.
